# Radiation injury

**Published:** 2010-09

**Authors:** Sameek Bhattacharya

**Affiliations:** Assistant Professor, Post Graduate Institute of Medical Education and Research, Dr. R.M.L. Hospital, New Delhi – 110 001, India

The incidence of radiation injury is not known because the cases are under-reported and the published reports are scattered. Radiation injuries have been reported from nuclear accidents, following radiation therapy and in healthcare personnel handling radiation and radioisotopes.[1] Plastic surgeons rarely encounter acute radiation injury but they do come across its delayed sequels, such as non-healing ulcers. These wounds pose a serious reconstructive challenge, as they are obstinate and prone to repeated breakdown after reconstructive procedure.[2]

In order to understand the radiation injury, it is important to go back to its basics. To quantify the amount of radiation exposure, the unit gray (Gy) is used, where 1 Gy denotes deposition of 1 J of energy per kilogram of tissue. But in order to set radiological protection standards, another unit Sievert (Sv) is used, which takes into account the biological effect of radiation. One Gray of beta or gamma radiation has 1 Sv of biological effect, whereas 1 Gy of alpha particle has 20 Sv effect. As the unit is large, doses related to humans are measured in millisieverts (mSv). International Commission on Radiological Protection (ICRP) guidelines state that the current maximum permissible dose to radiation workers is 20 mSv per year averaged over five years, with a maximum of 50 mSv in any one year.[3]

Harmful radiations are categorized as: alpha (α), beta (β) and gamma (γ) rays. Alpha particles are positively charged by Helium ions. These heavy particles can only travel a few centimetres in air and are unable to penetrate the keratin layer of skin. But these are high-energy particles with high Sv value and can cause extensive tissue damage when ingested or inhaled. Beta particles are negatively charged electron beams, which can travel several meters in air and can cause shallow injuries like sunburn, as they have limited penitrability (1 cm). Gamma rays, from X-rays and natural decay of radioisotopes like ^60^Co and ^192^Ir, can travel several meters in air and can penetrate deep into the tissues. Hence gamma rays can produce very deep damage involving vital structures like bone marrow and lungs. In addition to deep gamma burns on the skin, these patients have systemic involvement manifesting as Acute Radiation Syndrome (ARS).

The biological effect of ionizing radiation at the cellular level can be a direct blow on the DNA template compromising the structure and function of the cells. Consequently, the cells may die, stop dividing or get dysplastic leading to malignancy. The indirect damage is mediated by free radicals produced by ionization of water molecules. These free radicals either damage cells directly or affect the DNA in the nucleus. The fast dividing cells like lymphocytes, bone marrow cells, keratinocytes and intestinal mucosal cells are highly susceptible to radiation damage. The clinical presentation depends upon whether the exposure is to the whole body or only localized to a particular part. More than 1 Gy exposure of gamma rays to the whole body can lead to an ARS. The condition is preceded by a period of prodromal nausea and vomiting. Acute Radiation Syndrome involves most of the vital system of the body and with increasing exposure can manifest as hematopoietic disorder, gastrointestinal disturbance like diarrhoea, cardiovascular instability leading to hypotension and finally convulsion and motor disturbance when CNS is affected. The severity of local damage to the skin also follows the dose increment of exposure. An exposure of 4 Gy can result in only allopecia, erythema can appear at 10–15Gy and non-healing ulceration at 25 Gy. Blistering and necrosis can manifest at three weeks when the exposure is 30–50Gy but can manifest within one week if the dose is above 100 Gy.[4] The severity of cutaneous involvement can be assessed by surface ultrasound (7.5 Hz), Tc^99^ vascular scintigraphy, CT scan and MRI. Histopathological examination of skin can reveal vascular and inflammatory changes.

Ir-192 is a man-made radioisotope, which emits gamma radiation on decay. It is used in brachytherapy and in industrial radiography. There are reported injuries related to accidental exposure to radiation from ^192^Ir used in brachytherapy.[5] External exposure can cause burns, radiation poisoning, and death. High-energy gamma radiation from ^192^Ir also increases the risk of cancer. The immediate effect of such ionising radiation is at the cellular level due to direct damage to DNA, leading to cell death and cessation of proliferation of the surviving damaged cells. The long-term consequence of radiation is attributed to progressive fibrosis, vasculitis and obliterative endarteritis. This leads to dermal and subcutaneous tissue fibrosis and vascular compromise. The fibrosis is presumed to be mediated by proinflammatory and profibrotic cytokines such as tumour necrosis factor-alpha.[6] There is concomitant fibroblast dysfunction and depletion with decreased collagen deposition in soft tissues explaining the compromised healing function.[6] Since ^192^Ir emits highly penetrating gamma rays, it damages deeper tissues, which at times is difficult to fathom. The resultant fibrosis and vascular damage is progressive, hence its extent is not easy delineated. These factors explain the recurrent ulcerations and development of new ones at distant sites. The affected tissues remain compromised over time and the vascular status is never restored to normalcy. We have seen radiation ulcer on chest wall and groin after ten years of post-radiotherapy. Although the case profile presented by the authors had a single exposure, such clinical picture is more commonly seen with subacute injury due to recurrent exposure, which is quite probable in personnels handling radioisotopes.

A similar case of multiple and recurrent radiation ulcerations on hands was presented to us. He happened to be an orthopaedic surgeon, who had been operating under image intensifier for a decade. The devascularisation was so profound that he lost two distal phalanges due to osteoradionecrosis. Similar osteoradionecrosis is also seen in mandible after radiotherapy of head and neck.[6] The vascular compromise in these sub-acute and repeated exposures is more pronounced than after a single exposure. Histopathology of such affected tissues demonstrates microvascular changes such as replacement of smooth muscle with collagen, degeneration of elastic lamina, breakdown of endothelial lining and luminal thrombi. This vascular compromise not only poses healing and reconstructive challenge but also explains the pain, which is due to hypoxia. Since compromised irradiated tissues are also prone to infection, most of the involved bones develop osteomyelitis and are difficult to salvage. But there are reports of prevention of osteoradionecrosis by hyperbaric oxygen therapy.[7,8] The other major concern in these wounds is malignancy. All the excised tissues must be histopathologically examined with a high level of suspicion.

Medical management on outpatient basis suffices for mild cutaneous burns involving less than 10% total body surface area (TBSA). Such injuries resulting from exposure of less than 20 Gy manifest as erythema, oedema and desquamation. They are treated by topical anti-inflammatory, anti-proliferative, non-atrophogenic glucocorticoids, anti-histaminics, linoleic acid creams, tretinoin cream, non-adherent dressings, analgesics and systemic and local antibiotics as per sensitivity.[9] Moderate injuries following exposure of more than 40 Gy and involving 10–40% TBSA manifest with erythema, oedema, necrosis and ulceration. In addition to medical management, they require admission for debridement and reconstruction.[9] Severe radiation injuries following exposure of more than 400 Gy affecting more than 40% TBSA present with oedema, bullae and necrosis. In addition to the extensive skin damage, these patients also present with multisystemic ARS. Hence these patients need multispeciality intensive care involving intensivist, health physicist, haematologist and reconstructive surgeon.[9] Since the extent of radiation damage evolves over a period of time, it is prudent to wait till the whole extent is evident. The initial management should be limited to life-saving measures and supportive treatment.

The thumb rule of surgical management of radiation ulcer is to excise the whole radiation-affected tissue and reconstruct with a flap. Any attempt to address only the ulcer, that is only ‘tip of the ice berg’,is fraught with failure. Since the radiation damage is progressive, one must err towards the normal tissue while excising. But many a times the exact demarcation may not be obvious or the damage may be so extensive that complete excision may expose vital structures. In both these scenarios, some irradiated tissues are left behind leading to recurrence of ulcer. The reconstructive aspect is equally demanding. Most of the time, tissue adjacent to the ulcer is damaged by radiation, precluding the use of local or regional flaps. I am sure the authors were fortunate in successfully using a local transposition flap for the wrist ulcer. Even planning an islanded Littler’s flap from the radiation-affected hand could have been disastrous. A fasciocutaneous, muscle or a musculocutaneous flap from a distance is generally successful.[8] It is also preferable to plan a non-parasitic and complete inset of the flap in single-stage (as the TFL and LD used by the authors) wherein there is no need to divide the pedicle at a later stage. This form of reconstruction not only maintains good vascularity but also provides a source of healthy peri-vascular fibroblasts, which is sparse in the irradiated area.[10] Although there are reports regarding poor patency of microvascular anastomosis in irradiated area, free flaps have been used successfully in reconstructing these defects.[11,12] As long as the flap pedicle is anastomosed to a healthy recipient vessel at a distance, in non-irradiated area free flap is a safe option. In addition to the surgical options, there is a renewed interest in reviving the damaged fibroblast to potentiate the healing capacity of the wound. Autologous fat grafting provides adipose-derived adult stem cells, which may differentiate into healthy fibroblasts.[13] There are reports regarding local treatment of radiation ulcers with transforming growth factor -beta (TGFβ) and fibroblast growth factor (FGF) to improve the fibroblast function and wound contraction.[14]
